# Spatial Clusters of Condyloma Acuminata and the Regional Risk Factors in South Korea: Bayesian Spatial Regression Analysis

**DOI:** 10.2196/76751

**Published:** 2025-11-03

**Authors:** Joonsu Jang, Seyul Park, Byung Chul Chun

**Affiliations:** 1 Department of Preventive Medicine, College of Medicine, Korea University, 73 Goryeodae-ro, Seong-buk Gu, Seoul, 02841, Republic of Korea, 82 222861169

**Keywords:** sexually transmitted infections, condyloma acuminata, cluster analysis, spatial epidemiology, social determinants

## Abstract

**Background:**

Condyloma acuminata (CA), the clinical manifestation of infection with low-risk human papillomaviruses 6 and 11, is a common sexually transmitted infection (STI) with recurrent lesions and notable psychosocial and health system burden. Recent evidence indicates a substantial global burden, with prevalence ranging from 0.5% to 33.1% and incidence ranging from 24 to 2940 per 100,000 person-years, varying by age, sex, time, and geography. In South Korea, national insurance data show sustained increases in patients receiving care for CA during 2010 to 2019. Beyond individual behaviors, spatial proximity and contextual factors can produce clustered STI risk. However, the municipal-level spatial distribution of CA in Korea and its contextual correlates remain understudied.

**Objective:**

This study aimed to identify high-risk geographic clusters of CA in South Korea and determine the regional factors associated with its incidence rates.

**Methods:**

We conducted an ecological analysis using 2019 municipal-level data from the National Health Insurance Service of Korea. Spatial autocorrelation of CA incidence rates was evaluated using Moran’s I, and clustering was assessed with Getis-Ord Gi* to detect high-risk clusters. We then analyzed potential regional determinants using two Bayesian spatial regression models: the intrinsic conditional autoregressive model and the Besag–York–Mollié model. Key municipal-level variables included health behaviors, socioeconomic indicators, health care access, adult entertainment venue density, and risk of sexual violence. Results are reported as adjusted relative risks (aRRs) with 95% credible intervals (CrIs).

**Results:**

A total of 52,009 CA cases were identified in 2019, 70.03% (36,421/52,009) of which were in men. We found significant positive spatial autocorrelation in CA incidence rates (Moran’s I>0, *P*<.001), indicating nonrandom spatial clustering. The Getis-Ord Gi* analysis revealed several high-incidence clusters (*hotspots*) in metropolitan and southeastern regions of South Korea. In the Bayesian spatial models, higher CA incidence rates were associated with a greater share of the municipal budget spent on social welfare (aRR 1.005, 95% CrI 1.001‐1.009), a higher percentage of single-person households (aRR 1.034, 95% CrI 1.025‐1.043), and more adult entertainment establishments per 10,000 people (aRR 1.006, 95% CrI 1.001‐1.012).

**Conclusions:**

We identified significant geographic hotspots of CA and several community-level risk factors driving these patterns in South Korea. These findings highlight the importance of spatial surveillance and targeted public health interventions in high-risk areas. Adapting STI prevention programs to address local social determinants may help reduce the spread of CA in the identified hotspots.

## Introduction

### Background

Condyloma acuminata (CA), the clinical manifestation of infection with low-risk human papillomaviruses 6 and 11, remains a persistent public health concern due to its high incidence, recurrent course, psychosocial burden, and nontrivial health system costs [1]. Although generally asymptomatic, CA lesions can itch, bleed, and cause irritation or discomfort in the anogenital region [2]. Recent studies indicate that CA remains a substantial global burden, with prevalence ranging from 0.5% to 33.1% and incidence rates ranging from 24 to 2940 per 100,000 person-years, varying by age, sex, time, and geography [3]. In South Korea, population-based analyses using the Korean National Health Insurance Service (KNHIS) data showed sustained increases in patients receiving care for CA during 2010‐2019 [4]. Earlier Health Insurance Review and Assessment Service in South Korea data (2007‐2015) also documented rising prevalence and economic burden at the national level [5].

Several individual-level risk factors for CA have been identified in prior studies. These include early age at first sexual intercourse, engaging in sexual activity with multiple partners, unprotected intercourse [6], a history of other sexually transmitted infections (STIs) [7], and smoking tobacco [8]. However, most of these studies focused on individual behaviors and did not examine how regional or community-level factors contribute to the geographical heterogeneity of CA incidence rates.

Beyond person-to-person transmission, spatial proximity effects can produce geographically clustered STI patterns through several ecological mechanisms. Evidence from large urban settings demonstrated that exposure to high-STI neighborhoods increases infection risk in both geographically proximate and socially connected areas through spillover [9]. Also, municipalities that share contextual determinants such as urbanicity, socioeconomic deprivation, and access to sexual health services tend to exhibit correlated STI risks over space and time. Recent spatial studies of STIs demonstrate these mechanisms across settings, including spatiotemporal clustering of HIV in China with strong associations to urbanization and health-system indicators, and persistent hot spots of curable STIs in subnational analyses [10]. Korean data also showed spatial clustering for STI, underscoring that geographically structured ecological determinants can shape STI risk beyond individual behavior [11]. These mechanisms justify spatial analyses to identify clustered risk and its contextual determinants.

Despite the increasing CA burden, evidence on the municipal-level spatial distribution of CA in South Korea remains limited. Specifically, no study has quantified municipal-level spatial clustering of CA in South Korea and examined how local contextual factors relate to CA incidence rate. Filling this gap is essential for geographically targeted prevention and service planning.

### Objectives

We hypothesized that (1) CA incidence rates would cluster spatially (hot spots) and (2) area-level determinants would partially explain this clustering. To address these, we aimed to quantify municipal-level clustering of CA in South Korea and identify which regional characteristics are associated with higher CA incidence rates to inform geographically targeted interventions.

## Methods

### Study Design and Setting

We conducted a cross-sectional ecological study at the municipal level in South Korea for the year 2019. Reporting followed the STROBE (Strengthening the Reporting of Observational Studies in Epidemiology) checklist for cross-sectional studies (Checklist 1). The unit of analysis was the Si-Gun-Gu (municipalities, including cities, counties, and districts), of which there are 250 nationwide. By focusing on area-level data across all municipalities, this design allowed us to assess geographic variations and identify municipal-level risk factors associated with CA incidence rates in a single year.

### Data Sources and Variables

This study analyzed nationwide health insurance claims generated from the KNHIS. The KNHIS insures approximately 97% of residents, with the remainder covered by the Medical Aid program [12]. The KNHIS claims constitute almost complete national dataset for epidemiologic research in Korea. The claims files include dates of service, diagnosis and procedure codes, provider type and location, and basic beneficiary characteristics needed for population-level analyses; personally identifiable information is not available to investigators [13]. Because this was a secondary analysis of collected data, there was no clinical recruitment or patient enrollment.

The primary outcome was the incidence rates of CA in each municipality during 2019. CA cases were identified using the *International Classification of Diseases, 10th Revision, Clinical Modification* code A63.0 recorded in the principal or additional diagnosis fields. To define incident cases for 2019, first A63.0-coded claim was selected in 2019 as the index event. To avoid counting follow-up care from the same clinical episode as new incidents, a 30-day reattendance window was applied: any subsequent A63.0-coded claims occurring ≤30 days after the index claim were considered part of the same episode and not counted as additional incident events. The incident events were assigned to the municipality recorded in the claims and aggregated across the 250 municipalities of South Korea for 2019. Annual municipal incidence rates per 100,000 people were computed using midyear resident-registry denominators from Statistics Korea [14].

We assembled a broad set of municipal characteristics as potential risk factors for CA, drawing on multiple data sources. These variables encompassed several domains including health behaviors, welfare-related variables, health conditions, socioeconomic variables, health care accessibility, adult entertainment establishments, and the risk of sexual violence (Table S1 in Multimedia Appendix 1). Additionally, two demographic covariates were included to adjust for population structure: the sex ratio and the median age of the population in each municipality. These adjustments account for differences in the age and gender distribution that might influence CA incidence rates.

All municipal-level variables were obtained from authoritative national data sources for the year 2019 to ensure consistency with the outcome data. Health behavior, health condition, and welfare and socioeconomic indicators were extracted from the 2019 Korean Community Health Survey and the Korean Statistical Information Service databases [14,15]. The number of adult entertainment establishments in each area was gathered from the government’s open data portal [16], which provides official public data on local businesses and facilities. The regional risk of sexual violence was derived from a report published by the Korea Institute of Criminology and Justice [17]. By integrating these diverse data sources, we compiled a comprehensive dataset of regional variables hypothesized to be associated with CA incidence rates.

### Spatial Autocorrelation

The global Moran’s I statistics for the incidence rates of CA across the 250 municipalities were calculated to examine whether the geographic distribution of CA incidence rates exhibited spatial autocorrelation [18]. Moran’s I is a measure of global spatial autocorrelation that ranges from –1 to +1, where values close to +1 indicate strong clustering of similar values and values close to –1 indicate spatial dispersion of values. A value near 0 would indicate that CA incidence rates are spatially random. A spatial weights matrix was constructed based on the K-nearest neighboring municipalities for each area (K=4), using great-circle distances between municipality centroids to define neighbors. The K-nearest neighbors approach ensures that every municipality has a consistent number of neighboring areas considered, even for those with no direct geographic borders. Given this weights matrix, Moran’s I was computed for the CA incidence rates per 100,000 population, and a permutation test with 999 Monte Carlo simulations was conducted to determine the significance of the observed Moran’s I value. The null hypothesis for this test is that CA incidence rates are distributed randomly in space; a significant Moran’s I (*P*<.05) would indicate that spatial autocorrelation is present, justifying the use of spatial modeling techniques in subsequent analyses [19].

### Cluster Detection (Hotspot Analysis)

In addition to global autocorrelation, we explored local clusters of high or low CA incidence rates using the Getis-Ord Gi* statistic [20]. The Getis-Ord Gi* is a local spatial statistic that identifies *hotspots* and *coldspots*, meaning areas where the incidence rates are significantly higher or lower than would be expected by chance, given the values in neighboring areas. For each municipality, Gi* considers the value of CA incidence rates in that area and those of its surrounding neighbors (using K=4) and computes a *z* score. A high positive *z* score with a significant *P* value (<.05) indicates a clustering of high incidence rates (a hotspot), whereas a high negative *z* score indicates a clustering of low incidence rates (a coldspot). Municipalities with a Gi* statistic exceeding the 95% CI bounds were mapped as significant clusters of elevated or reduced CA incidence rates. This hotspot analysis complements the global Moran’s I by identifying where spatial clusters occur, providing insight into specific high-risk regions.

### Variable Selection and Nonspatial Regression Analysis

Prior to building spatial models, we conducted a nonspatial regression analysis to identify important predictors of CA incidence rates and to ensure that our model would account for overdispersion in the count data [21]. Initially, a Poisson regression framework was considered because the number of CA cases in each municipality is a count outcome. However, we tested for overdispersion in the count data by comparing the variance to the mean and using an overdispersion test statistic [22]. The overdispersion test indicated that the variance of CA cases across municipalities was significantly greater than the mean (*P*<.05). We therefore chose a negative binomial regression model to account for the overdispersion.

Using the negative binomial framework, bivariate regression analyses were performed for each candidate independent variable. In these models, the outcome was the count of CA cases in the municipality, and the log of the municipality’s population was included as an offset term. Each candidate variable was entered individually to assess its crude association with CA incidence rates. A liberal significance criterion of *P*≤.25 was used to screen for potential predictors [23]. In other words, any variable that showed an association with CA incidence rates at *P*≤.25 in the bivariate analysis was retained for consideration in the multivariable model. This cutoff is more inclusive than the conventional .05 level and was chosen to avoid prematurely excluding variables that might become significant in a multivariable context [23].

Next, a multivariable negative binomial regression model was used, including all candidate variables that passed the screening threshold. Variance inflation factors were computed for the covariates to check for multicollinearity, with a variance inflation factor >5 used as an indicator of high multicollinearity that could distort the model estimates [24]. If any pair of variables were highly collinear, we planned to remove or combine one of them, favoring the variable with more direct relevance to CA or the one that yielded the better model fit. The final nonspatial model was selected through a backward elimination procedure. Starting with the full set of selected covariates, we iteratively removed the least significant variable one at a time and compared model fit using the Akaike Information Criterion. The final model provided a baseline set of risk factors for CA incidence rates without accounting for spatial effects.

### Bayesian Spatial Modeling

We incorporated spatial dependence into our regression modeling using a Bayesian approach because the exploratory spatial analysis revealed evidence of nonrandom spatial clustering in CA incidence rates. A Bayesian hierarchical spatial modeling was adopted because the outcome comprises municipal counts exhibiting overdispersion and residual spatial autocorrelation. Traditional nonhierarchical spatial regression often struggles to accommodate these characteristics in small-area count settings due to the absence of area-level random effects to capture residual dependence [25]. The Bayesian framework enables us to specify a negative binomial likelihood with a population offset, incorporate area-level spatial random effects that borrow strength from neighboring municipalities, and quantify uncertainty with posterior intervals.

Two types of Bayesian spatial regression models were implemented to account for residual spatial autocorrelation in the incidence rates of CA across municipalities. These were the intrinsic conditional autoregressive (ICAR) model and the Besag–York–Mollié (BYM) model [26]. Both models are commonly used in disease mapping to improve estimates by *borrowing strength* from neighboring areas and to control for spatially structured confounding [27]. The ICAR model introduces a spatially structured random effect that assumes nearby areas have similar risk, whereas the BYM model includes two random effect components—one spatially structured and one unstructured—allowing it to capture both localized spatial clustering and independent heterogeneity at the municipal level.

The municipal incidence rates of CA were modeled using a negative binomial likelihood. We let *Y_i_* denote the counts of CA in municipality *i* (*i*=1,…,250).


(1) $$Y_{i}∼\;NB\left(r,\;p_{i}\right),\;\;p_{i}=\frac{r}{r+\lambda_{i}},\;\;E\left(Y_{i}\right)=\lambda_{i},\;\;Var\left(Y_{i}\right)=\lambda_{i}+\frac{\lambda_{i}^{2}}{r}$$

Here, *r*>0 is the size (overdispersion) parameter and *p_i_* is the success probability. The mean $\lambda_{i}$ is linked to covariates with a population offset:


(2) $$\log\left(\lambda_{i}\right)=\alpha +\log\left(N_{i}\right)+\sum_{k}\beta_{k}X_{i,k}+\eta_{i}$$

where α is the intercept, *N_i_* is the municipal population (offset), *X_i,k_* are municipal-level covariates with coefficients $\beta_{k}$, and $\eta_{i}$ is a spatial random effect. To account for residual spatial autocorrelation, we first specified $\eta_{i}$ under an ICAR prior, which imposes spatial smoothing such that neighboring municipalities have similar risk:


(3) $$\eta_{i}=s_{i},\;\;s={\left(s_{1},\;\ldots ,\;s_{250}\right)}^{T}∼\;ICAR\;\left(W,\;\sigma_{s}^{2}\right)$$

The spatial weights matrix *W* was built using K=4 K-nearest neighbors computed from municipal centroids in projected coordinates. We then considered the BYM extension, which augments the ICAR component with an independent, spatially unstructured term to capture residual heterogeneity:


(4) $$\eta_{i}=s_{i}+u_{i},u_{i}\sim N(0,\sigma_{u}^{2})$$

Both ICAR and BYM models are widely used in disease mapping to borrow strength across adjacent areas and to mitigate spatially structured confounding [28,29]. We fitted both ICAR and BYM specifications and compared performance using deviance information criterion (DIC) values with practical and theoretical considerations. For regression outputs, the posterior median of the exponentiated fixed effects was reported as an adjusted relative risk (aRR) with a 95% credible interval (CrI). For clarity, in the Bayesian framework, a 95% CrI denotes the range containing 95% of the posterior probability mass for the parameter, given the model and prior. Covariate effects were considered significant when the 95% CrI for the aRR does not include 1.00.

The posterior marginal distribution of the model parameters was approximated using the integrated nested Laplace approximation [30]. A flat prior distribution was applied to the intercept α and the regression parameters β corresponding to each explanatory variable and covariate. For the variance associated with spatially structured and spatially unstructured terms, a weakly informative prior known as the Penalized Complexity prior was applied [31].

We conducted a sensitivity analysis to examine the robustness of the findings to the choice of spatial neighborhood structure. The primary analysis defined spatial adjacency using each municipality’s four nearest neighbors (K=4). For sensitivity testing, the Bayesian spatial model was rerun under two alternative neighbor definitions: first using three nearest neighbors (K=3) and then using five nearest neighbors (K=5) for each municipality. All the results were compared across the different neighbor specifications: spatial autocorrelation statistics, identified hotspots, and key regression coefficients. The sensitivity analysis confirmed that the overall spatial patterns and the identified significant predictors of CA incidence rates remained consistent when using K=3 or K=5, suggesting that our findings are robust to reasonable variations in the spatial neighborhood definition.

All statistical analyses were performed using R version 4.3.1 (R Foundation for Statistical Computing). Spatial autocorrelation and clustering statistics (global Moran’s I and Getis-Ord Gi*) were computed with the *spdep* package [32]; all maps were produced with *tmap* [33]; the nonspatial negative binomial baseline model was fitted using *MASS* [34]; overdispersion was evaluated with *AER* [22]; and Bayesian spatial models were estimated with *R-INLA* [30]. The shape file was obtained from the National Spatial Geographic Information Service [35].

### Ethical Considerations

This study was conducted using aggregated, deidentified health data and publicly available municipal statistics. The Institutional Review Board of Korea University reviewed and approved this study (IRB approval number: KUIRB-2021-0382-01).

## Results

### Descriptive Statistics

In 2019, a total of 52,009 cases of CA were recorded across the 250 municipalities, with approximately 70.03% (36,421/52,009) of cases in men. Across municipalities, the mean number of CA cases was 207.3 (SD 179.5) per municipality, and the mean CA incidence rate was 95.8 per 100,000 people (Table 1). The burden of CA varied widely between regions: the number of cases ranged from as low as 3 to as high as 886 in different municipalities, and incidence rates ranged from 1.46 to 184.21 per 100,000 people. This represents more than a 100-fold difference in incidence rates across communities, highlighting substantial geographic disparities in CA occurrence. The coefficient of variation for CA cases was 0.87, reflecting this high relative dispersion. By comparison, the potential regional risk factors examined showed coefficients of variation ranging from 0.01 up to 0.85, indicating that none of these variables exhibited as much regional variability as the incidence rates of CA.

**Table 1.** Descriptive statistics for condyloma acuminata (CA; *ICD-10-CM*^a^ A63.0) and potential regional risk factors across 250 municipalities examined in this nationwide cross-sectional ecological study of South Korea (2019).^b^

		Mean (SD)	Minimum	Median	Maximum	CV^c^
Cases of CA, n		207.26 (179.48)	3	159.50	886	0.87
Incidence rates of CA (per 100,000 people)		95.83 (27.08)	1.46	95.04	184.21	0.28
Health behavior						
	Exercise and physical activities (%)	25.36 (6.88)	5.50	24.75	56.90	0.27
	Current smoking (%)	20.27 (3.09)	11.60	20.25	28.80	0.15
	Alcohol consumption (%)	59.28 (4.36)	44.80	59.90	71.00	0.07
	High-risk drinking (%)	14.16 (2.98)	5.80	14.10	24.80	0.21
Welfare-related variables						
	Social welfare facilities per 100,000 people	19.12 (11.83)	2.30	16.65	73.70	0.62
	Share of municipal budget on social welfare (%)	34.70 (14.71)	11.40	32.90	66.60	0.42
	Subjective health perception (%)	42.64 (6.98)	29.70	41.40	68.30	0.16
	EQ-5D index	0.96 (0.01)	0.93	0.96	0.99	0.01
	Healthy living practice (%)	29.02 (9.48)	9.60	28.50	54.70	0.33
Health conditions						
	Prevalence of depression (%)	3.04 (1.38)	0.40	2.90	7.20	0.45
	Prevalence of obesity (%)	34.38 (3.72)	24.30	34.25	44.80	0.11
	Prevalence of diabetes (%)	8.04 (1.32)	5.00	8.00	11.80	0.16
	Prevalence of hypertension (%)	19.44 (2.28)	14.90	19.30	27.40	0.12
Socioeconomic variables						
	Low educational attainment (%)	51.39 (11.65)	14.84	52.32	73.13	0.23
	Single-person household (%)	23.90 (5.50)	12.99	23.80	39.05	0.23
	Divorce per 1000 people	2.18 (0.42)	1.20	2.20	4.10	0.19
	Financial autonomy (%)	25.16 (14.01)	7.05	20.93	68.90	0.56
Health care accessibility						
	Doctors per 1000 people	2.78 (2.20)	1.00	2.30	19.60	0.79
	Unmet medical facilities (%)	6.48 (3.40)	0.90	6.00	19.10	0.52
Adult entertainment and sexual violence						
	Adult entertainment establishments per 10,000 people	7.70 (6.53)	0.11	6.32	64.29	0.85
	Sexual violence risk	99.95 (18.22)	68.53	96.22	203.78	0.18
Covariates						
	Median age (y)	47.05 (6.03)	36.80	45.55	61.00	0.13
	Sex ratio (%)	101.53 (6.72)	87.60	100.40	134.70	0.07

a *ICD-10-CM*: *International Classification of Diseases, 10th Revision, Clinical Modification.*

b The table summarizes municipal-level determinants alongside annual CA cases and incidence rates per 100,000 people.

c CV: coefficient of variance.

### Spatial Clustering

**Figure 1.**
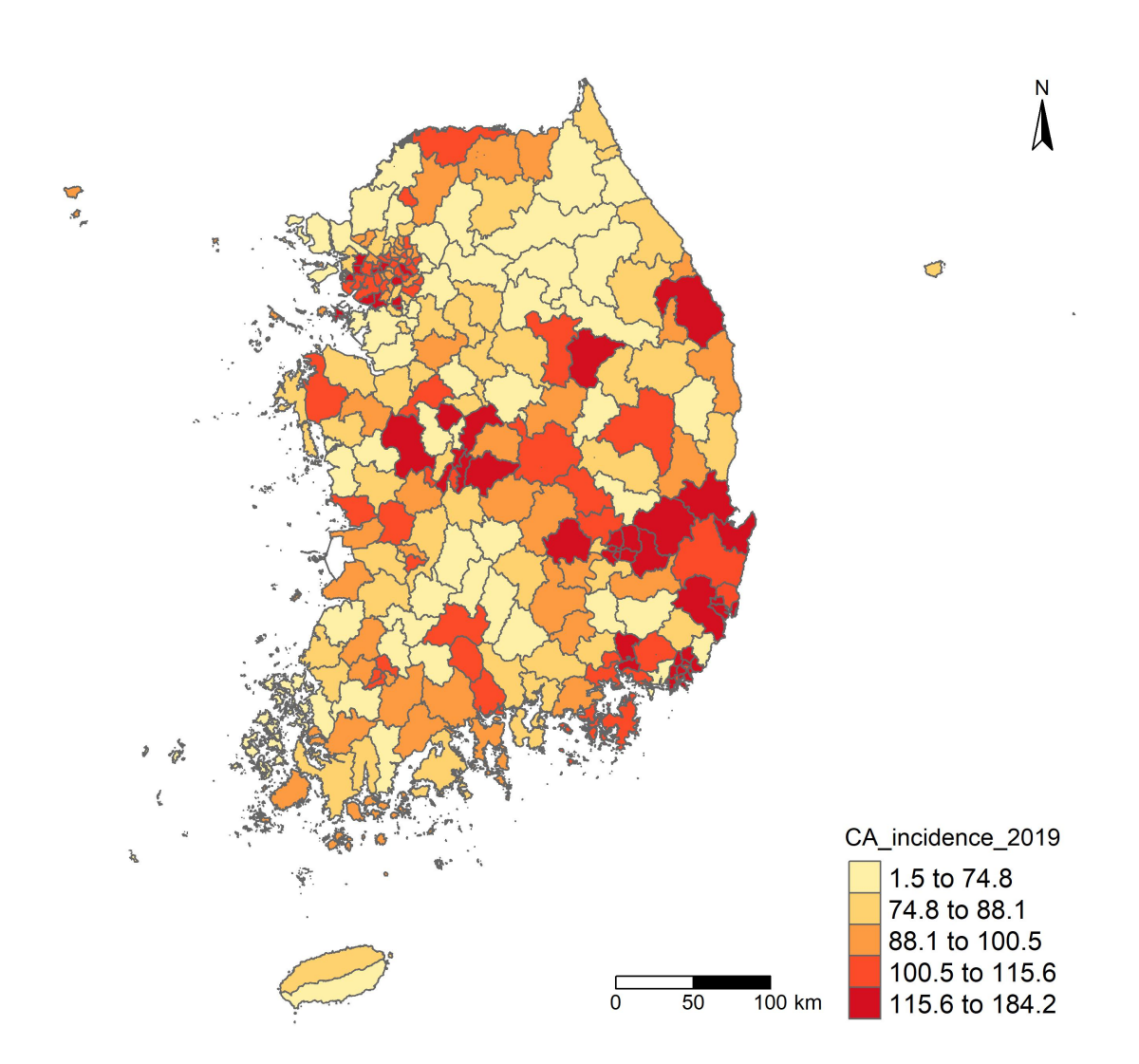
Geographic distribution of condyloma acuminata (CA; *International Classification of Diseases, 10th Revision, Clinical Modification* A63.0) incidence rates per 100,000, by municipality (n=250), in this nationwide cross-sectional ecological study of South Korea (2019). Incident CA cases were aggregated to the municipality of residence; denominators are midyear resident registry populations. The choropleth shades each municipality by CA incidence rates. Lighter to darker tones indicate lower to higher incidence rates.

**Figure 2.**
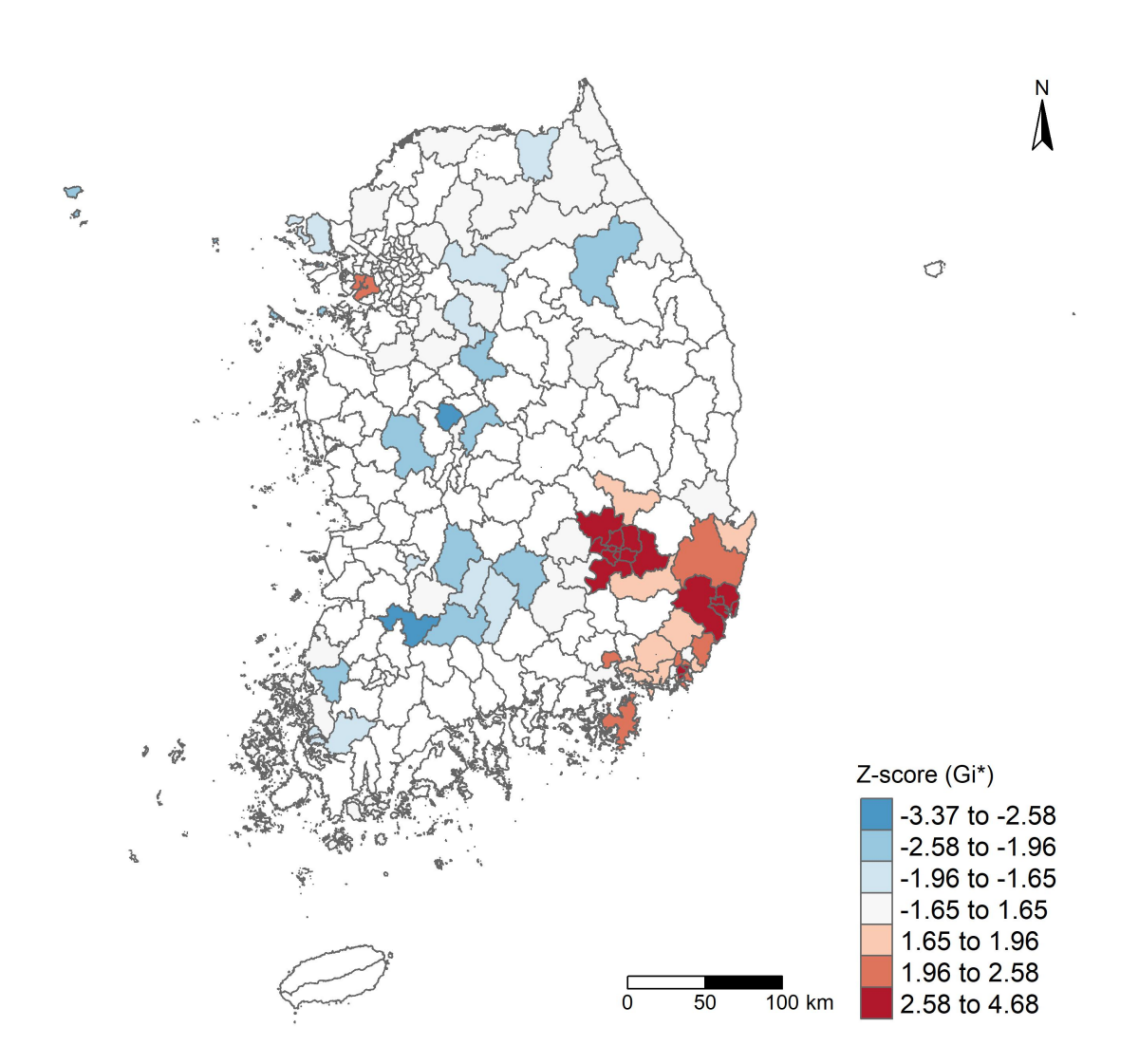
Hotspot and coldspot clustering of condyloma acuminata (CA; *International Classification of Diseases, 10th Revision, Clinical Modification* A63.0) incidence rates per 100,000 based on the Getis–Ord Gi* analysis at the municipality (n=250) level for South Korea (2019). Spatial weights were defined by K-nearest neighborhood contiguity (K=4). Municipalities are colored by Gi* *z* score: statistically significant hotspots are shown in red tones (clusters of high incidence rates), and statistically significant coldspots are shown in blue tones (clusters of low incidence rates); nonsignificant areas are shown in white. Significance thresholds are |*z*|≥1.96 (*P*<.05) and |*z*|≥2.58 (*P*<.01).

The choropleth map of CA incidence rates (Figure 1; Multimedia Appendix 2) indicated that municipalities in certain metropolitan and southeastern parts of South Korea showed higher incidence rates compared to those in the northeastern and southwestern regions, where rates were lower. Consistent with these visual patterns, the Global Moran’s I analysis confirmed significant positive spatial autocorrelation. Specifically, the incidence rates of CA were spatially clustered across municipalities (Moran’s I for incidence rates=0.335, *P*<.001), as were the case counts (Moran’s I=0.395, *P*<.001). Local cluster detection using the Getis-Ord Gi* statistic further identified specific areas of high and low incidence rates. The Gi* analysis identified several significant hotspots of increased CA incidence rates in the urbanized metropolitan area and the southeastern region (Figure 2).

### Regression Modeling

After the preliminary nonspatial analysis and variable selection procedure, five candidate variables were selected for the spatial regression modeling: social welfare facilities per 100,000 people, share of municipal budget on social welfare (%), percentage of single-person households, prevalence of hypertension (%), and number of adult entertainment establishments per 10,000 people. In addition, two demographic covariates, median age and sex ratio, were included in the models to adjust for population structure. Model fit was similar for the ICAR and BYM specifications (ICAR: DIC 2422.38; BYM: DIC 2421.36), with ∆DIC=1.02. While the DIC difference was minimal, we selected the BYM model for theoretical reasons. The BYM model accounts for both structured spatial dependence and unstructured spatial heterogeneity, providing a more comprehensive representation of spatial effects. The BYM model decomposed the total spatial variance into structured (precision=101.48, 95% CI 34.84‐250.05) and unstructured components (precision=17,485.45, 95% CI 202.07‐119,072.66), indicating the presence of both spatial clustering and municipality-specific variation. Therefore, we present the results from the BYM model as the final spatial regression model (Table 2). The ICAR model yielded nearly identical fixed effect estimates, with magnitudes of fixed effect, directions, and statistical significance remaining consistent across both models, demonstrating that our findings are robust to spatial model specification.

The Bayesian spatial regression identified three key regional factors that were significantly associated with higher CA incidence rates after adjusting for covariates and spatial effects. First, the share of the municipal budget devoted to social welfare showed a positive association: for each 1% increase in welfare budget share, the aRR of CA increased to 1.004 (95% CrI 1.001‐1.008), suggesting a small but significant increase in risk. Second, the proportion of single-person households in a municipality was strongly associated with CA incidence rates: each 1% increase in single-person households corresponded to the aRR of 1.033 (95% CrI 1.024‐1.041). Third, the density of adult entertainment establishments was linked to higher CA incidence rates: for each additional adult entertainment establishment per 10,000 population, the aRR was 1.006 (95% CrI 1.001‐1.012).

Sensitivity analyses confirmed the robustness of the spatial regression findings. We refit the Bayesian models using alternative spatial neighborhood definitions. The model fit remained best when using K=4 neighbors, as evidenced by the lowest DIC at K=4 compared to K=3 or K=5. Importantly, the direction and magnitude of the associations for the identified risk factors were consistent across these different spatial weights (Table S2 in Multimedia Appendix 1).

**Table 2.** Association between municipal-level determinants and incidence rates of condyloma acuminata (*ICD-10-CM*^a^A63.0) in this nationwide cross-sectional ecological study of South Korea (2019).

	Nonspatial model (multiple negative binomial regression)^b^	Spatial models^c^	
		ICAR^d^	BYM^e^
Social welfare facilities per 100,000 people, aRR^f^ (95% CrI^g^)	0.998 (0.994‐1.001)	0.998 (0.995‐1.002)	0.998 (0.995‐1.002)
Share of municipal budget on social welfare (%), aRR (95% CrI)	1.006 (1.003‐1.010)	1.005 (1.001‐1.008)	1.004 (1.001‐1.008)
Single-person household (%), aRR (95% CrI)	1.030 (1.022‐1.039)	1.032 (1.024‐1.041)	1.033 (1.024‐1.041)
Prevalence of hypertension (%), aRR (95% CrI)	0.987 (0.973‐1.002)	0.997 (0.981‐1.013)	0.998 (0.983‐1.014)
Adult entertainment establishments per 10,000 people, aRR (95% CrI)	1.008 (1.003‐1.013)	1.006 (1.001‐1.012)	1.006 (1.001‐1.012)
Median age (y), aRR (95% CrI)	1.019 (1.011‐1.027)	1.014 (1.006‐1.023)	1.014 (1.005‐1.022)
Sex ratio (%), aRR (95% CrI)	1.008 (1.002‐1.014)	1.005 (0.999‐1.011)	1.004 (0.998‐1.010)
Dispersion parameter (*r*), posterior median (95% CrI)	22.518 (17.825‐27.974)	27.799 (20.853‐36.094)	29.022 (21.620‐38.022)
Precision of spatially structured component ($\tau_{s}$), posterior median (95% CrI)	—^h^	160.414 (42.191‐478.918)	101.485 (34.842‐250.054)
Precision of spatially unstructured component ($\tau_{u}$), posterior median (95% CrI)	—	—	17,485.451 (202.075‐119,072.658)
DIC^i^	2440.852	2422.379	2421.362

a *ICD-10-CM*: *International Classification of Diseases, 10th Revision, Clinical Modification.*

b Nonspatial generalized regression model.

c Two Bayesian spatial models with municipal adjacency based on K-nearest neighborhood contiguity (K=4).

d ICAR: intrinsic conditional auto-regressive model.

e BYM: Besag–York–Mollié model.

f aRR: adjusted relative risk (fixed effect).

g CrI: credible interval.

h Not applicable.

i DIC: deviance information criterion.

## Discussion

### Principal Findings

This study revealed geographic disparities in the incidence rates of CA across South Korea. A total of 52,009 CA cases were analyzed, and the municipal-level incidence rates ranged from as low as 1.46 to 184.21 per 100,000 people—over a 100-fold difference between the lowest and highest areas (Table 1). Such variation indicates substantial spatial heterogeneity in CA burden. Indeed, we found significant positive global spatial autocorrelation (Moran’s I=0.33, *P*<.001), confirming that municipalities with high CA incidence rates tended to cluster near each other. The choropleth map of CA incidence rates (Figure 1) and the Getis-Ord Gi* hotspot analysis (Figure 2) consistently identified distinct high-risk clusters in the urbanized metropolitan regions and the southeastern part of the country. These hotspots emphasize that the transmission of CA is not distributed evenly but rather concentrated in certain communities at an increased risk. In addition to mapping clusters, our analysis identified key regional factors associated with the spatial distribution of CA. Using Bayesian spatial regression modeling, we found three significant municipal-level risk factors that remained positively associated with higher CA incidence rates after adjusting for demographic covariates and spatial autocorrelation (Table 2). First, municipalities that spent a higher share of their budget on social welfare programs had a higher relative risk of CA. Second, areas with a higher proportion of single-person households showed increased CA incidence rates. Third, municipalities with more adult entertainment establishments per 10,000 people had higher CA rates. The Bayesian model that included both spatially structured and unstructured random effects—the BYM model—provided a better fit than the purely spatial ICAR model, indicating that accounting for local heterogeneity in addition to broad spatial trends improved the explanation of CA incidence rates patterns.

Our findings of spatial clustering of CA incidence rates are consistent with patterns observed in other settings. The spatial autocorrelation observed in CA incidence rates does not result from direct person-to-person transmission between neighboring individuals but rather from the geographic clustering of municipal-level risk factors that create similar high-risk environments in spatially proximate areas. Previous geographic studies have also identified localized hotspots for STIs. For example, mapping of STIs in Portugal found distinct high-rate clusters in certain regions [36], and a cross-sectional analysis reported that per-capita STI incidence rates tended to increase systematically with urban population size [37]. Likewise, cross-setting analyses report nonrandom spatial clustering of STIs that aligns with contextual determinants such as urbanicity, socioeconomic conditions, and access to sexual health services [9,38]. In our study, municipalities with higher proportions of single-person households and greater density of adult entertainment establishments exhibited a higher incidence of CA, suggesting that local sexual market structures and alcohol-related social environments may influence opportunities for risky behavior [39]. Furthermore, the highest CA incidence rates were found in densely populated urban and southeastern areas, which is consistent with other studies and suggests that large urban centers often serve as hubs for STI transmission. This aligns with the concept of core transmission areas, where densely interconnected sexual networks facilitate wider spread of infection [40]. Our results support this concept, as the identified metropolitan clusters may act as reservoirs that support wider disease spread. A similar spatial concentration of STIs has been demonstrated in the United States, where county-level analyses using hotspot methods have identified significant clusters of increased STI prevalence [41]. Both our study and prior research suggest that geographic context, especially urbanicity and regional connectivity, plays a central role in STI dynamics.

The positive association we observed between the prevalence of single-person households and CA incidence rates is supported by epidemiological data on sexual behavior. Single individuals are more likely to have multiple sexual partners compared to married or cohabiting individuals [42], and having multiple partners is a well-established risk factor for acquiring CA and other STIs [43]. A Turkish study similarly noted that unmarried status was linked to higher risk of human papillomavirus infection [44]. Thus, areas with many one-person households may inherently have higher levels of casual or transient sexual partnering in the population, contributing to greater CA transmission. Our findings tentatively extend this individual-level evidence to the community level, indicating that municipalities with a higher proportion of single-person households also tend to have higher incidence rates of CA.

The finding that municipalities with more adult entertainment establishments have higher CA incidence rates is also consistent with previous research. Such establishments may foster networks of high-risk sexual contacts. Studies in the United States have documented that individuals working in or around the adult entertainment industry have a higher STI prevalence and engage in riskier sexual behaviors than the general population [45,46]. These studies, combined with our findings, point to adult entertainment establishments as important sites of STI transmission. They likely attract or employ core groups with higher numbers of sexual encounters, thereby amplifying the spread of infection in the surrounding community. Our findings reinforce the need for targeted public health intervention in such areas to reduce local transmission of CA.

In contrast, the positive association we found between higher social welfare expenditure and CA incidence rates appears to be a novel observation with no direct precedent in the literature. This counterintuitive finding should be interpreted with caution. One plausible explanation is reverse causation or confounding: municipalities facing a high burden of CA may allocate a higher share of their budget to social services and public health, including STI management and prevention programs. In other words, increased welfare spending may be a response to the existing public health needs rather than a causal driver of infection. Alternatively, this association may reflect underlying social deprivation regions with need for welfare support could simultaneously have higher STI rates, as suggested by broader public health studies linking low socioeconomic status to increased STI prevalence [47]. CA is a condition requiring long-term management with a tendency for recurrence [48]. Communities heavily impacted by recurrent CA cases may incur higher health care and social support costs, prompting larger welfare budgets. Since no prior study, to our knowledge, has examined this factor in relation to CA, our result highlights a new avenue for research. Further studies are needed to disentangle whether higher social welfare spending is a marker of underlying social determinants that facilitate CA spread or whether it reflects an intensified local response to control an ongoing epidemic [49].

### Limitations

This study has limitations. First, the analysis was conducted at the municipal level using an ecological design. All data, including CA cases and risk factors, were aggregated by municipality; individual-level exposures and behaviors could not be assessed. Consequently, the associations we identified do not necessarily imply causation at the individual level, and they may be susceptible to the ecological fallacy. Second, the cross-sectional nature of our study limits our ability to infer temporal trends or causality. We captured a one-year snapshot of CA incidence rates and regional characteristics, but the situation may evolve over time. Third, our reliance on the NHIS records for CA cases means the data are subject to any inconsistencies in diagnosis or reporting. Genital warts carry social stigma [50], which could lead to underdiagnosis or underreporting in certain areas. For instance, individuals might avoid seeking treatment for an STI due to shame, or clinicians might use nonspecific diagnostic codes to spare patient discomfort. Such variability in health care–seeking behavior and diagnostic practices across municipalities could introduce measurement bias in the recorded incidence rates. Fourth, while we included a wide range of regional covariates, it is possible that unmeasured confounders also influence the spatial distribution of CA. Finally, the spatial resolution of our analysis was at the municipality level; intra-municipal variation or clustering at a finer neighborhood scale could not be examined with the available data. Despite these limitations, this study provides a foundation for addressing CA incidence rates through evidence-based strategies, focusing on identified hotspots and regional risk factors.

### Conclusions

In summary, our nationwide spatial analysis of CA in South Korea revealed significant geographic clustering, with hotspots concentrated in the metropolitan and southeastern regions, and demonstrated that area-level factors, particularly a higher prevalence of single-person households and greater density of adult entertainment establishments, are associated with increased incidence rates of CA. These findings highlight the value of integrating spatial epidemiology into routine surveillance to identify communities at greatest risk and to guide the efficient allocation of targeted interventions. Moreover, the observed association with social welfare expenditure suggests that policymakers should consider how recurrent STI burdens strain local resources and ensure that funding for prevention and care is targeted to the most affected areas. Future research should explore the causal pathways linking these regional characteristics to CA transmission and evaluate the impact of geographically targeted public health interventions over time.

## Supplementary material

10.2196/76751 Multimedia Appendix 1 Description and source of study variables and Bayesian spatial regression model results.

10.2196/76751 Multimedia Appendix 2 Regional distribution of condyloma acuminata (CA) cases in South Korea in 2019. Bright yellow shading represents a low number of CA cases, whereas red shading denotes a high number of CA cases.

10.2196/76751 Checklist 1 STROBE checklist.

## Abbreviations

aRR: adjusted relative risk
BYM: Besag–York–Mollié
CA: condyloma acuminata
CrI: credible interval
DIC: deviance information criterion
ICAR: intrinsic conditional autoregressive
KNHIS: Korean National Health Insurance Service
STI: sexually transmitted infection
STROBE: Strengthening the Reporting of Observational Studies in Epidemiology
