# Supplementary material for: Spatial Clusters of Condyloma Acuminata and the Regional Risk Factors in South Korea: Bayesian Spatial Regression Analysis
Source: JMIR Public Health Surveill. 2025 Nov 3;11:e76751. doi: 10.2196/76751 (PMC12582512; doi:10.2196/76751)
Supplement: Multimedia Appendix 1 [file publichealth-v11-e76751-s001.docx]

**Table S1**. Description and source of study variables.

| Variables | Description | Source (year) |
| --- | --- | --- |
| Condyloma acuminata cases | Condyloma acuminata (A630, ICD-10) patients in 2019 by municipality | NHIS^a^ (2019) |
| Health behavior |  |  |
| Exercise and physical activities | Percentage of people who engaged in moderate physical activity that made them feel a little more tired or short of breath than usual, at least 30 minutes a day, five days a week, in the past week, per municipality. | KCHS^b^ (2019) |
| Current smoking | Percentage of current smokers ("daily smokers" or "occasional smokers") who have smoked 5 or more packs (100 cigarettes) in their lifetime (so far) per municipality. | KCHS (2019) |
| Alcohol consumption | Percentage of people who drank alcohol at least once a month in the past year per municipality. | KCHS (2019) |
| High-risk drinking | Percentage of people who drank 7 or more drinks (or about 5 cans of beer) on a single occasion for men and 5 or more drinks (or about 3 cans of beer) for women at least twice a week in the past year per municipality. | KCHS (2019) |
| Welfare related variables |  |  |
| Social welfare facilities per 100,000 people | Number of social welfare facilities per 100,000 people per municipality. | KOSIS^c^ (2019) |
| Share of municipal budget on social welfare | Percentage of total budget for social welfare and health in the current year per municipality. | KOSIS (2019) |
| Subjective health perception | Percentage of people who say their health is "very good" or "good" on a typical day per municipality. | KCHS (2019) |
| EQ-5D index | European Quality of Life-5 Dimensions: an index that synthesizes a technical system of five dimensions of health-related quality of life (mobility, self-care, daily activities, pain/discomfort, and anxiety/depression) per municipality. | KCHS (2019) |
| Healthy living practice | Percentage of people who smoke, drink alcohol, and walk per municipality. | KCHS (2019) |
| Health Conditions |  |  |
| Prevalence of depression | Percentage of people with a total depression screening tool (PHQ-9) score of 10 or higher walk per municipality. | KCHS (2019) |
| Prevalence of obesity | Percentage of people with a BMI (kg/m^2^) of 25 or higher per municipality. | KCHS (2019) |
| Prevalence of diabetes | Percentage of people aged 30 and older who have been diagnosed with diabetes by a doctor per municipality. | KCHS (2019) |
| Prevalence of hypertension | Percentage of people aged 30 and older who have been diagnosed with high blood pressure by a doctor per municipality. | KCHS (2019) |
| Socio-economic variables |  |  |
| Low educational attainment | Percentage of people aged 6 and older with a high school diploma or less as their highest level of education per municipality. | KOSIS (2015) |
| Single-person household | Percentage of people in single-person household by municipality. | KOSIS (2019) |
| Divorce per 1,000 people | Number of divorces in a year divided by the mid-year population in that year per 1,000 people per municipality. | KOSIS (2019) |
| Financial autonomy (%) | Total earned income over total expense, per municipality. | KOSIS (2019) |
| Healthcare accessibility |  |  |
| Doctors per 1,000 people | Number of doctors (medical doctors, Korean medicine doctors, dentists) working in health care organizations per 1,000 people, per municipality. | KOSIS (2019) |
| Unmet medical facilities | Percentage of people who were unable to go to the doctor (excluding dentist) when they wanted to in the past year per municipality. | KCHS (2019) |
| Adult entertainment and sexual violence |  |  |
| Adult entertainment establishments per 10,000 people | Number of establishments per 10,000 population that employ entertainment workers or have entertainment facilities, such as stand bars, room salons, and karaoke clubs, where food is prepared and sold primarily with alcoholic beverages. | Public data portal (2019)  (https://www. data.go.kr/) |
| Sexual violence risk | Risk of sexual violence using Crime Risk Assessment Tool as applied to 250 municipalities. | Park et al. (2015) [1] |
| Covariates |  |  |
| Median age | Median age per municipality. | KOSIS (2019) |
| Sex ratio | Number of men per 100 women per municipality. | KOSIS (2019) |
| Offset |  |  |
| Population | Mid-year population based on the resident registry per municipality | KOSIS (2019) |

^a^NHIS: National Health Insurance Service

^b^KCHS: Korea Community Health Survey

^c^KOSIS: Korea Statistical Information Service

**Table S2**. Bayesian spatial regression model results on risk factors for the incidence of condyloma acuminata per 100,000 people at the municipal level in South Korea in 2019, comparing k-nearest neighbor matrices with k=3, 4, and 5. The results were expressed as relative risks (95% credible intervals).

|  | k as 3 | | k as 4 | | k as 5 | |
| --- | --- | --- | --- | --- | --- | --- |
|  | **ICAR^a^** | **BYM^b^** | **ICAR** | **BYM** | **ICAR** | **BYM** |
| Social welfare facilities per 100,000 population | 0.998 (0.995–1.001) | 0.998 (0.995–1.002) | 0.998 (0.995–1.002) | 0.998 (0.995–1.002) | 0.998 (0.995–1.001) | 0.998 (0.995–1.002) |
| Share of municipal budget on social welfare (%) | 1.005 (1.001–1.009) | 1.005 (1.001–1.008) | 1.005 (1.001–1.008) | 1.004 (1.001–1.008) | 1.005 (1.001–1.008) | 1.004 (1.001–1.008) |
| Single-person household (%) | 1.032 (1.024–1.040) | 1.032 (1.024–1.041) | 1.032 (1.024–1.041) | 1.033 (1.024–1.041) | 1.032 (1.024–1.041) | 1.033 (1.024–1.041) |
| Prevalence of hypertension (%) | 0.996 (0.981–1.012) | 0.998 (0.982–1.014) | 0.997 (0.981–1.013) | 0.998 (0.983–1.014) | 0.997 (0.980–1.013) | 0.998 (0.982–1.014) |
| Adult entertainment establishments per 10,000 population | 1.006 (1.001–1.012) | 1.006 (1.001–1.012) | 1.006 (1.001–1.012) | 1.006 (1.001–1.012) | 1.006 (1.001–1.012) | 1.006 (1.001–1.012) |
| Median age (years of age) | 1.014 (1.006–1.023) | 1.014 (1.005–1.022) | 1.014 (1.006–1.023) | 1.014 (1.005–1.022) | 1.014 (1.005–1.023) | 1.014 (1.005–1.022) |
| Sex ratio (%) | 1.005 (0.999–1.011) | 1.004 (0.998–1.010) | 1.005 (0.999–1.011) | 1.004 (0.998–1.010) | 1.005 (0.999–1.012) | 1.005 (0.999–1.011) |
| DIC^c^ | 2422.721 | 2423.179 | 2422.379 | 2421.362 | 2423.221 | 2422.131 |

^a^ICAR: intrinsic conditional auto-regressive model

^b^BYM: Besag-York-Mollie model.

^c^DIC: deviance information criterion

**Reference**

1 Park J, Kang Y, Kim D, et al. The development of crime risk assessment tool and its application in South Korea (Ⅲ) – with focus on sexual violence and school violence-: Korean Institute of Criminology, 2014.
