# Supplementary figures and images for: Spatial Clusters of Condyloma Acuminata and the Regional Risk Factors in South Korea: Bayesian Spatial Regression Analysis
Source: JMIR Public Health Surveill. 2025 Nov 3;11:e76751. doi: 10.2196/76751 (PMC12582512; doi:10.2196/76751)

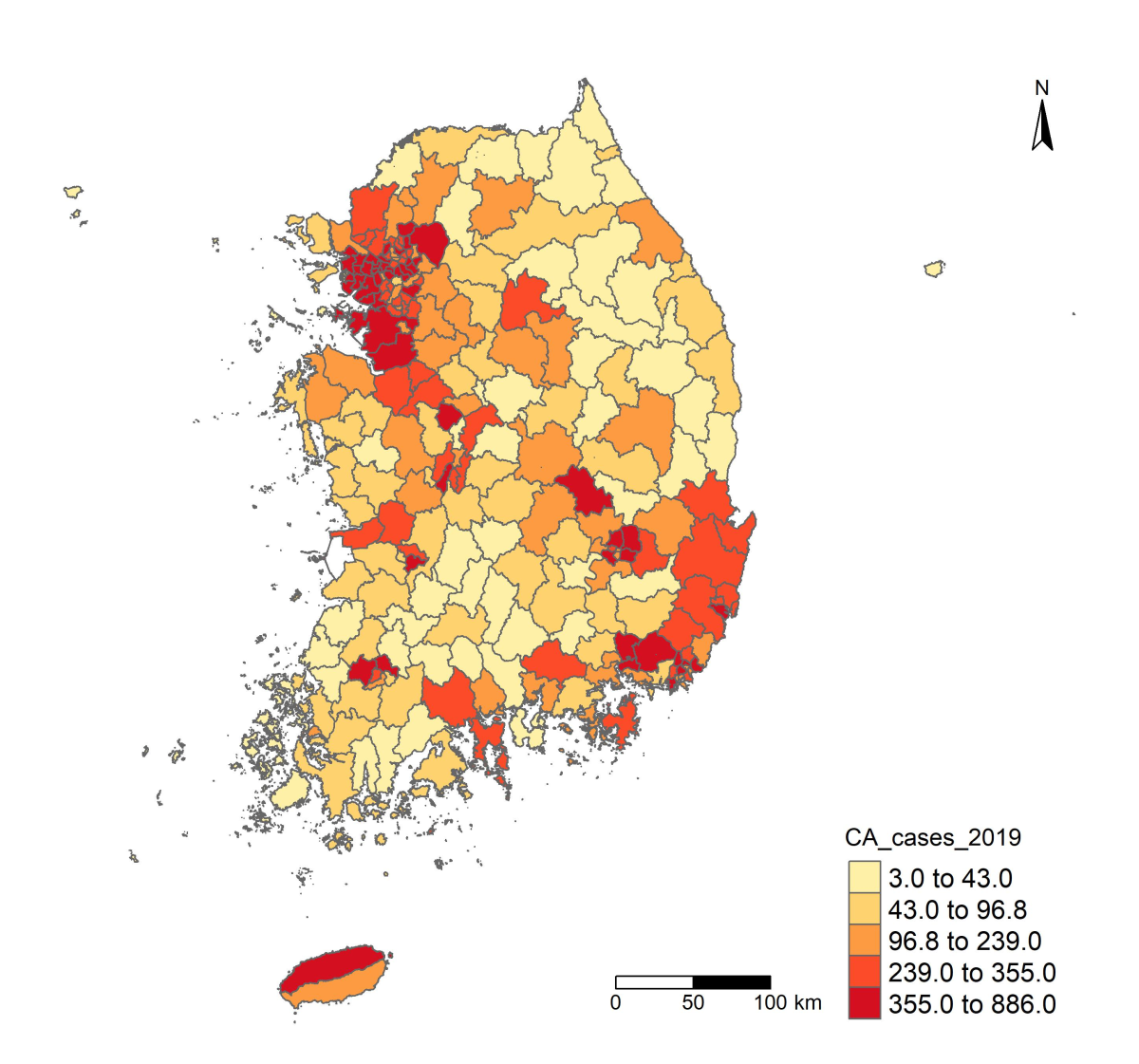

Supplement: Multimedia Appendix 2 [file publichealth-v11-e76751-s002.png]
